# Poly[μ-bromido-μ-(2,2-dimethyl­propane-1,3-diyl diisocyanide)-silver(I)]: a powder diffraction study

**DOI:** 10.1107/S1600536810044703

**Published:** 2010-11-13

**Authors:** Mahmoud Al-Ktaifani, Mwaffak Rukiah

**Affiliations:** a Department of Chemistry, Atomic Energy Commission of Syria (AECS), PO Box 6091, Damascus, Syrian Arab Republic

## Abstract

In the title compound, [AgBr(C_7_H_10_N_2_)]_*n*_, adjacent Ag(I) atoms are bridged by bidentate CNCH_2_C(CH_3_)_2_CH_2_NC ligands *via* the NC groups, forming [Ag{CNCH_2_C(CH_3_)_2_CH_2_NC}]_*n*_ chains with the metal atom in a distorted tetrahedral coordination. The bromide counter-anions cross-link the Ag(I) atoms of the chains, forming a two-dimensional polymeric network {[Ag^I^(CNCH_2_C(CH_3_)_2_CH_2_NC)]Br}_*n*_ extend­ing parallel to (010). The polymeric structure is similar to that of the very recently reported Cl^−^, I^−^ and NO_3_^−^ analogues. This gives a strong indication that 2,2-dimethyl­propane-1,3-diyl diisocyanide is a potential ligand for giving polymeric structures on treatment with Ag*X* (*X* = Cl^−^, Br^−^, I^−^ or NO_3_^−^) regardless of the counter-anion used.

## Related literature

For the preparation of the bidentate ligand CNCH_2_C(CH_3_)_2_CH_2_NC, see: Al-Ktaifani *et al.* (2008 ▶). For similar polymeric structures, see: Al-Ktaifani *et al.* (2008 ▶); Rukiah & Al-Ktaifani (2008 ▶, 2009 ▶). For disocyano ligands and their coordination complexes, see: Harvey (2001 ▶); Sakata *et al.* (2003 ▶); Espinet *et al.* (2000 ▶); Moigno *et al.* (2002 ▶). For chelate complexing, see: Chemin *et al.* (1996 ▶). Pseudo-Voigt profile coefficients as parameterized in Thompson *et al.* (1987 ▶). Asymmetry correction of Finger *et al.* (1994 ▶). Microstrain broadening by Stephens (1999 ▶). Indexing was performed using the program *DICVOL04* (Boultif & Louër, 2004 ▶). The best estimated space group was determined with the help of the program *Check Group* inter­faced by *WinPLOTR* (Roisnel & Rodriguez-Carvajal, 2001 ▶). The powder diffraction pattern was subsequently refined using the LeBail method by the program *FULLPROF* (Rodriguez-Carvajal, 2001 ▶). The program *GSAS* (Larson & Von Dreele, 2004 ▶) was inter­faced by *EXPGUI* (Toby, 2001 ▶). The preferred orientation was modeled using a spherical-harmonics description (Von Dreele, 1997 ▶).
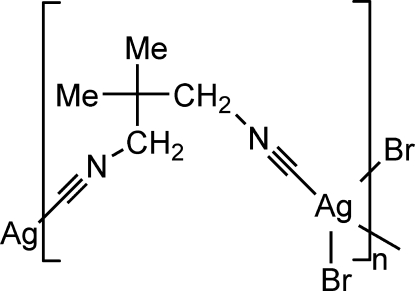


## Experimental

### 

#### Crystal data

[AgBr(C_7_H_10_N_2_)]
*M* *_r_* = 309.94
Orthorhombic, 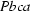

*a* = 16.24649 (13) Å
*b* = 16.59379 (12) Å
*c* = 7.40433 (4) Å
*V* = 1996.14 (2) Å^3^
*Z* = 8
Cu *K*α_1_ radiation, λ = 1.54060 Å
μ = 20.43 mm^−1^
*T* = 298 K
flat sheet, 7.0 × 7.0 mm

#### Data collection

STOE STADI P Transmission diffractometer
Specimen mounting: drifted powder between two Mylar foils
Data collection mode: transmission
Scan method: step
Absorption correction: for a cylinder mounted on the ϕ axis function for a flat sample in transmission geometry (*GSAS*; Larson & Von Dreele, 2004 ▶) *T*_min_ = 0.139, *T*_max_ = 0.192
2θ_min_ = 4.97°, 2θ_max_ = 89.95°, 2θ_step_ = 0.02°

#### Refinement

*R*_p_ = 0.020
*R*_wp_ = 0.027
*R*_exp_ = 0.021
*R*(*F* ^2^) = 0.019
χ^2^ = 1.638
4250 data points
172 parameters
40 restraints
H-atom parameters constrained

### 

Data collection: *WinXPOW* (Stoe & Cie, 1999 ▶); cell refinement: *GSAS* (Larson & Von Dreele, 2004 ▶); data reduction: *WinXPOW*; program(s) used to solve structure: *FOX* (Favre-Nicolin & Černý, 2002 ▶); program(s) used to refine structure: *GSAS* (Larson & Von Dreele, 2004 ▶); molecular graphics: *ORTEP-3* (Farrugia, 1997 ▶); software used to prepare material for publication: *publCIF* (Westrip, 2010 ▶).

## Supplementary Material

Crystal structure: contains datablocks global, I. DOI: 10.1107/S1600536810044703/er2080sup1.cif

Rietveld powder data: contains datablocks I. DOI: 10.1107/S1600536810044703/er2080Isup2.rtv

Additional supplementary materials:  crystallographic information; 3D view; checkCIF report

## Figures and Tables

**Table:** 

Ag1—Br1	2.7680 (19)
Ag1—Br1^i^	2.832 (2)
Ag1—C1	2.140 (9)
Ag1—C7^ii^	2.162 (10)

**Table:** 

Br1—Ag1—Br1^i^	106.50 (7)
Br1—Ag1—C1	107.1 (4)
Br1—Ag1—C7^iii^	98.9 (4)
Br1^i^—Ag1—C1	106.9 (4)
Br1^i^—Ag1—C7^iii^	94.6 (4)
C1—Ag1—C7^iii^	139.3 (6)
Ag1—Br1—Ag1^iv^	93.22 (6)

Symmetry codes: (i) 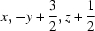; (ii) 
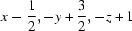
; (iii) 
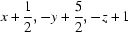
; (iv) 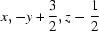.

**Fig. 1.**
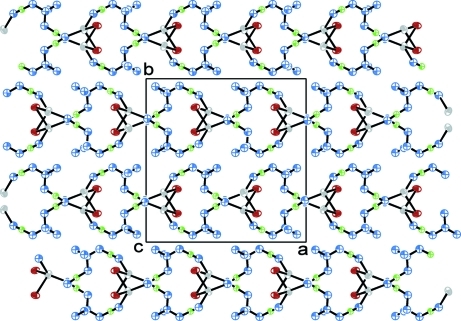
A view, along the c axis of the crystal structure of compound (I), H atoms are not shown for clarity.

**Fig. 2.**
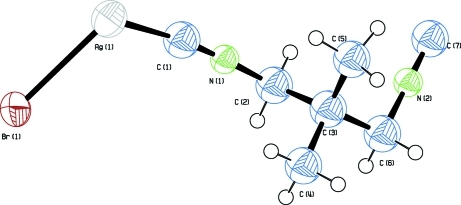
The asymmetric unit of (I) and atom-numbering are shown.

**Fig. 3.**
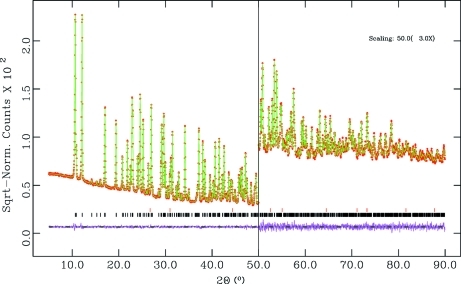
Final observed (points), calculated (line) and difference profiles for the Rietveled refinement of (I).

## supplementary crystallographic information

### Comment

In order to better understand and further explore the chemistry of 2,2-dimethylpropane-1,3-diyl diisocyanide, the synthesis and solid state characterization of the polymeric complex {[Ag^I^(CNCH_2_C(CH_3_)_2_CH_2_NC)]Br}_n_ is presented. Treatment of AgBr with two equimolar amount of 2,2-dimethylpropane-1,3-diyl diisocyanide in dry EtOH at room temperature afforded a highly insoluble white powder (I) even in polar or coordinate solvents. These strongly gave an indication that the obtained compound (I) have a polymeric structure, which is very similar to the polymeric structure of {[Ag^I^(CNCH_2_C(CH_3_)_2_CH_2_NC)]*X*}_n_ (*X* = Cl^-^ or I^-^) (Al-Ktaifani *et al.*, 2008; Rukiah & Al-Ktaifani, 2009).

The solid state structure of (I) was confirmed by X-ray powder diffraction study exhibiting, as expected, a polymeric structure, which is very similar to the analogous Cl^-^, I^-^ and NO_3_^-^ polymers. In the obtained structure, the Ag^I^ centers are bridged with each of the two adjacent Ag neighbours by the bidentate ligands CNCH_2_C(CH_3_)_2_CH_2_NC *via* the NC groups to form {Ag^I^(CNCH_2_C(CH_3_)_2_CH_2_NC)}_n_ chains. The Br^-^ counterpart anions are cross linked the Ag centres of the chains to form a polymeric 2-D network {[Ag^I^(CNCH_2_C(CH_3_)_2_CH_2_NC)]Br}_n_ (Fig. 1). In the same manner to the polymeric structure of Cl^-^, I^-^ and NO_3_^-^ analogues, the CNCH_2_C(CH_3_)_2_CH_2_NC in the complex just behaves as bis-monodentate and the chelate behaviour is completely absent. This is undoubtedly expected for steric reason as the distance between the two isocyanide groups in the CNCH_2_C(CH_3_)_2_CH_2_NC molecule are relatively too short to allow chelate complexing (Chemin *et al.*, 1996) (Fig.2).

As the conformation of the bidentate ligand (CNCH_2_C(CH_3_)_2_CH_2_NC) in the three polymeric structures are almost alike, it can be concluded that their molecular structures are very similar. Therefore it can be stated the counterpart anion (Cl^-^, Br^-^ or I^-^) have no effective role in changing the polymeric structure of the complex. It is also noteworthy, that the bidentate ligand exhibits a very strong tendency to form polymeric complexes rather than dimeric or trimeric complexes suggesting the 2,2-dimethylpropane-1,3-diyl diisocyanide to be a potential bidentate ligand in the syntheses of organometallic polymers of different transition metals.

### Experimental

All reactions and manipulations were carried out under inert atmosphere by using two fold vacuum line and schlenk technique. Solvents were dried and distilled over sodium wire; glassware dried and flamed before used. AgBr was a commercial sample and was used as received. IR spectra were operated on FTIR *Jasco 300* E. Microanalysis was performed using *EURO EA*. Powder X-ray diffraction was performed by *Stoe* Transmission diffractometre (*Stadi P*).

A solution of CNCH_2_C(CH_3_)_2_CH_2_NC (0.30 g, 2.45 mmol) in EtOH (5 ml) was added to a suspension of AgBr (0.22 g, 1.19 mmol) in dry EtOH (10 ml) at room temperature. The resulting solution was stirred for overnight, and then filtered and volatiles were removed *in vacuo*. The obtained product was washed with ether to afford a white powder (0.29 g, yield 80%, m.p. starts to decompose at 395 K). Analytical data for AgC_7_H_10_N_2_Br: found C, 27.95%; H, 3.35%; N, 7.99%; required: C, 27.12%; H, 3.25%; N, 9.03%. IR (KBr) νcm^-1^: 2201.6 (N≡ C).

### Refinement

The powder sample was slightly ground in a mortar, loaded into two foils of Mylar and fixed in the sample holder with a mask of suitable internal diameter (7.0 mm). Data were collected at room temperature and pressure in transmission geometry employing *Cu K_α__1 _*radiation. Indexing was performed using the program *DICVOL04* (Boultif & Louër, 2004) with default options. An orthorhombic unit cell of reasonable volume (assuming *Z*=8) gave indexing figures of merit *M*_20_=18.0, F_20_=36.9(0.0090, 60). The best estimated space group in the orthorhombic system was *Pcab* which determined with the help of the program *Check Group* interfaced by *WinPLOTR* (Roisnel & Rodriguez-Carvajal, 2001). The parameters *a* and *b* were interchanged for working with the standard setting of space group i.e *Pbca*. The powder diffraction pattern from 5 to 90° (2q) was subsequently refined with these cell and space group using LeBail method by the program *FULLPROF* (Rodriguez-Carvajal, 2001). One line with very low intensity was not indexed with the previous cell and corresponds to the reflection (111) of the AgBr. The program *FOX* (Favre-Nicolin & Černý, 2002) was employed for structure solution. The powder pattern was truncated to 55° in *2*θ (*Cu K_α__1 _*), corresponding to real-space resolution of 1.67 Å. The Monte Carlo simulated annealing (parallel tempering algorithm) used to solve the crystal structure of compound (I) from powder pattern in direct space. One molecule of CNCH_2_C(CH_3_)_2_CH_2_NC ligand and two free atoms of Ag and Br were introduced randomly in the orthorhombic cell calculated by Le Bail refinement. The H atoms can be ignored during the structure solution process because they do not contribute significantly to the powder diffraction pattern, due to their low X-ray scattering power. During the parallel tempering calculations, the ligand had the possibility to translate, to rotate around its centre of mass and to modify its torsion angles and the atoms Ag and Br had the possibility to modify its position in the unit cell. The model found by *FOX* was introduced in the program *GSAS* (Larson & Von Dreele, 2004), interfaced by *EXPGUI* (Toby, 2001) for Rietveld refinements as a starting point. The background was refined using a shifted Chebyshev polynomial with 20 coefficients. The Thompson-Cox-Hastings (Thompson *et al.*, 1987) pseudo-Voigt profile function was used with an axial divergence asymmetry correction of (Finger *et al.*, 1994). The two asymmetry parameters of this function *S/L* and *D/L* were both fixed at 0.0215 during the Rietveld refinement.

Geometric soft restraints were applied to the C°N, N—C and C—C distances to guide them towards their normal values, but no restrains were imposed on the Ag—C and Ag—Br distances. Likewise, no restraints were imposed on bond angles. The hydrogen atoms were introduced at theoretical positions with CH_2_ and CH_3_ distances constrained to be 0.97 Å for CH_3_ and 0.98 Å for CH_2_. They were refined with restrains on their bonds distances and bond angles to their normal values. One isotropic atomic displacement parameter was introduced per types of atoms C, N and H. The final refinement cycles were performed using anisotropic displacement parameters for Ag and Br atoms. Intensities were corrected for absorption effects with a function for a flat plate sample in transmission geometry (function number 4 in *GSAS*). The value of with m.d was 0.8. The plate normal can be either perpendicular to the diffraction vector or tilted by some fixed angle φ in the diffraction plane. The preferred orientation was modeled using a spherical-harmonics description (Von Dreele, 1997) with 18 coefficients. In the course of the refinement, the structure of AgBr has been introduced in the final refinement. The unit-cell parameters, the atomic displacement parameters of Ag and Br and the profile parameters were allowed to vary of this compound. The amount of this impurity was about 0.1%. The observed and calculated diffraction patterns for the refined crystal structure are shown in Fig. 3.

### Crystal data

**Table:**

[AgBr(C_7_H_10_N_2_)]	*F*(000) = 1184.0
*M**_r_* = 309.94	*D*_x_ = 2.063 Mg m^−^^3^
Orthorhombic, *P**b**c**a*	Cu *K*α_1_ radiation, λ = 1.54060 Å
Hall symbol: -P 2ac 2ab	µ = 20.43 mm^−^^1^
*a* = 16.24649 (13) Å	*T* = 298 K
*b* = 16.59379 (12) Å	Particle morphology: fine powder visual estimate
*c* = 7.40433 (4) Å	White
*V* = 1996.14 (2) Å^3^	flat sheet, 7.0 × 7.0 mm
*Z* = 8	Specimen preparation: Prepared at 298 K and 101.3 kPa

### Data collection

**Table:**

STOE STADI P Transmission diffractometer	Scan method: step
Radiation source: sealed X-ray tube, C-Tech	Absorption correction: for a cylinder mounted on the φ axis function for a flat plate sample in transmission geometry absorption correction '*GSAS* (Larson & Von Dreele, 2004)'
Ge 111	*T*_min_ = 0.139, *T*_max_ = 0.192
Specimen mounting: drifted powder between two Mylar foils	2θ_min_ = 4.97°, 2θ_max_ = 89.95°, 2θ_step_ = 0.02°
Data collection mode: transmission	

### Refinement

**Table:**

Least-squares matrix: full	Excluded region(s): none
*R*_p_ = 0.020	172 parameters
*R*_wp_ = 0.027	40 restraints
*R*_exp_ = 0.021	H-atom parameters constrained
*R*(*F*^2^) = 0.01907	(Δ/σ)_max_ = 0.02
χ^2^ = 1.638	Background function: GSAS Background function number 1 with 20 terms. Shifted Chebyshev function of 1st kind 1: 1519.26 2: -1446.56 3: 737.807 4: -254.400 5: 19.2069 6: 34.2759 7: -57.6962 8: 44.0465 9: 4.23667 10: 15.2262 11: -36.4418 12: 19.8416 13: 11.6080 14: -21.7772 15: 7.42188 16: 2.13196 17: -8.09916 18: 10.4475 19: -3.62142 20: 2.86699
4250 data points	Preferred orientation correction: Spherical harmonics function

### Special details

**Table:**

Experimental. The sample was ground lightly in a mortar, loaded between two Myler foils and fixed in the sample holder with a mask of 7.0 mm intrnal diameter.

### Fractional atomic coordinates and isotropic or equivalent isotropic displacement parameters (Å^2^)

**Table:**

	*x*	*y*	*z*	*U*_iso_*/*U*_eq_	
Ag1	0.38967 (9)	0.80095 (7)	0.4117 (2)	0.05958	
Br1	0.30684 (11)	0.82642 (9)	0.0900 (3)	0.06015	
C1	0.5098 (7)	0.7575 (9)	0.344 (2)	0.057 (2)*	
C2	0.6370 (3)	0.6752 (3)	0.2513 (6)	0.057 (2)*	
C3	0.6320 (4)	0.5873 (3)	0.3162 (7)	0.057 (2)*	
C4	0.5598 (2)	0.5488 (2)	0.2127 (5)	0.057 (2)*	
C5	0.6150 (2)	0.5772 (2)	0.5193 (5)	0.057 (2)*	
C6	0.7112 (3)	0.5453 (3)	0.2551 (6)	0.057 (2)*	
C7	0.8331 (7)	0.6053 (8)	0.4282 (19)	0.057 (2)*	
N1	0.5660 (5)	0.7181 (6)	0.3133 (11)	0.044 (3)*	
N2	0.7802 (6)	0.5754 (5)	0.3512 (12)	0.044 (3)*	
H2a	0.68677	0.70043	0.30004	0.1*	
H2b	0.63872	0.67661	0.11895	0.1*	
H6a	0.70639	0.48721	0.27692	0.1*	
H6b	0.71937	0.55477	0.12573	0.1*	
H4a	0.57835	0.53189	0.09379	0.1*	
H4b	0.53985	0.50232	0.2793	0.1*	
H4c	0.51576	0.5878	0.19985	0.1*	
H5a	0.6622	0.596	0.58768	0.1*	
H5b	0.56685	0.60861	0.5522	0.1*	
H5c	0.60499	0.52086	0.54582	0.1*	

### Atomic displacement parameters (Å^2^)

**Table:**

	*U*^11^	*U*^22^	*U*^33^	*U*^12^	*U*^13^	*U*^23^
Ag1	0.0490 (14)	0.0637 (13)	0.0660 (12)	0.0106 (10)	0.0053 (16)	−0.0066 (11)
Br1	0.079 (2)	0.0452 (15)	0.0560 (16)	0.0074 (12)	−0.011 (2)	0.0034 (14)

### Geometric parameters (Å, °)

**Table:**

Ag1—Br1	2.7680 (19)	C3—C5	1.538 (5)
Ag1—Br1^i^	2.8322 (22)	C3—C6	1.533 (5)
Ag1—C1	2.140 (9)	C4—H4a	0.97
Ag1—C7^ii^	2.162 (10)	C4—H4b	0.97
Br1—Ag1	2.7680 (19)	C4—H4c	0.97
Br1—Ag1^iii^	2.8322 (22)	C5—H5a	0.97
C1—N1	1.146 (16)	C5—H5b	0.97
C2—C3	1.538 (5)	C5—H5c	0.97
C2—N1	1.431 (5)	C6—H6a	0.98
C2—H2a	0.98	C6—H6b	0.98
C2—H2b	0.98	C7—Ag1^iv^	2.162 (10)
C3—C4	1.540 (5)	C7—N2	1.144 (16)
			
Br1—Ag1—Br1^i^	106.50 (7)	C3—C4—H4b	109.3
Br1—Ag1—C1	107.1 (4)	C3—C4—H4c	109.5
Br1—Ag1—C7^v^	98.9 (4)	H4a—C4—H4b	109.5
Br1^i^—Ag1—C1	106.9 (4)	H4a—C4—H4c	109.4
Br1^i^—Ag1—C7^v^	94.55 (35)	H4b—C4—H4c	109.4
C1—Ag1—C7^v^	139.3 (6)	C3—C5—H5a	109.4
Ag1—Br1—Ag1^iii^	93.22 (6)	C3—C5—H5b	109.4
Ag1—C1—N1	164.9 (14)	C3—C5—H5c	109.4
C3—C2—N1	109.2 (6)	H5a—C5—H5b	109.5
C3—C2—H2a	109.5	H5a—C5—H5c	109.5
C3—C2—H2b	109.7	H5b—C5—H5c	109.5
N1—C2—H2a	109.5	C3—C6—N2	110.8 (6)
N1—C2—H2b	109.4	C3—C6—H6a	109.3
H2a—C2—H2b	109.5	C3—C6—H6b	109.2
C2—C3—C4	106.1 (4)	N2—C6—H6a	109.1
C2—C3—C5	114.7 (4)	N2—C6—H6b	109.1
C2—C3—C6	107.1 (4)	H6a—C6—H6b	109.3
C4—C3—C5	107.76 (35)	Ag1^vi^—C7—N2	154.8 (13)
C4—C3—C6	107.7 (4)	C1—N1—C2	171.9 (13)
C5—C3—C6	113.0 (4)	C6—N2—C7	174.9 (13)
C3—C4—H4a	109.6		
			
C1—Ag1—Br1—Ag1^iii^	31.9 (5)	N1—C2—C3—C4	68.0 (13)
C7^ii^—Ag1—Br1—Ag1^iii^	−179.7 (4)	N1—C2—C3—C5	−50.8 (14)
Br1^i^—Ag1—Br1—Ag1^iii^	−82.21 (8)	N1—C2—C3—C6	−177.1 (10)
Br1—Ag1—C7^ii^—N2^ii^	70 (3)	C2—C3—C6—N2	68.7 (13)
C1—Ag1—C7^ii^—N2^ii^	−160 (3)	C4—C3—C6—N2	−177.4 (10)
Br1—Ag1—Br1^i^—Ag1^i^	−168.31 (7)	C5—C3—C6—N2	−58.6 (14)
C1—Ag1—Br1^i^—Ag1^i^	77.5 (4)		

Symmetry codes:  (i) *x*, −*y*+3/2, *z*+1/2; (ii) *x*−1/2, −*y*+3/2, −*z*+1; (iii) *x*, −*y*+3/2, *z*−1/2; (iv) *x*+1/2, −*y*+3/2, −*z*+1; (v) *x*+1/2, −*y*+5/2, −*z*+1; (vi) *x*+3/2, −*y*+5/2, −*z*+1.

### Hydrogen-bond geometry (Å, °)

**Table:**

*D*—H···*A*	*D*—H	H···*A*	*D*···*A*	*D*—H···*A*
C2—H2A···N2	0.98	2.60	2.950 (17)	101.0
C4—H4C···N1	0.97	2.45	2.908 (18)	108.0
C5—H5A···N2	0.97	2.62	2.959 (18)	101.0
C5—H5B···N1	0.97	2.53	2.903 (16)	103.0
C6—H6A···Br1^vii^	0.98	2.85	3.820 (13)	169.0
C6—H6B···Br1^viii^	0.98	2.91	3.671 (13)	136.0

Symmetry codes: (vii) *x*+1/2, −*y*+3/2, −*z*; (viii) −*x*+1, *y*−1/2, −*z*+1/2.
